# Unhealthy smokers: scopes for prophylactic intervention and clinical treatment

**DOI:** 10.1186/s12868-017-0388-6

**Published:** 2017-10-04

**Authors:** Shikha Prasad, Mohammad Abul Kaisar, Luca Cucullo

**Affiliations:** 1 Department of Neurology, Northwestern University Feinberg School of Medicine, Chicago, Illinois 60611 USA; 2 Department of Pharmaceutical Sciences, School of Pharmacy, Texas Tech University Health Sciences Center, 1300 S. Coulter Street, Amarillo, TX 79106 USA; 3 Center for Blood Brain Barrier Research, Texas Tech University Health Sciences Center, Amarillo, TX 79106 USA

**Keywords:** Environment, Inflammation, Epigenetic, Smoking cessation, Addiction, Intervention

## Abstract

**Background:**

Globally, tobacco use causes approximately 6 million deaths per year, and predictions report that with current trends; more than 8 million deaths are expected annually by 2030. Cigarette smokings is currently accountable for more than 480,000 deaths each year in United States (US) and is the leading cause of preventable death in the US. On average, smokers die 10 years earlier than nonsmokers and if smoking continues at its current proportion among adolescents, one in every 13 Americans aged 17 years or younger is expected to die prematurely from a smoking-related illness. Even though there has been a marginal smoking decline of around 5% in recent years (2005 vs 2015), smokers still account for 15% of the US adult population. What is also concerning is that 41,000 out of 480,000 deaths results from secondhand smoke (SHS) exposure. Herein, we provide a detailed review of health complications and major pathological mechanisms including mutation, inflammation, oxidative stress, and hemodynamic and plasma protein changes associated with chronic smoking. Further, we discuss prophylactic interventions and associated benefits and provide a rationale for the scope of clinical treatment.

**Conclusions:**

Considering these premises, it is evident that much detailed translational and clinical studies are needed. Factors such as the length of smoking cessation for ex-smokers, the level of smoke exposure in case of SHS, pre-established health conditions, genetics (and epigenetics modification caused by chronic smoking) are few of the criteria that need to be evaluated to begin assessing the prophylactic and/or therapeutic impact of treatments aimed at chronic and former smokers (especially early stage ex-smokers) including those frequently subjected to second hand tobacco smoke exposure. Herein, we provide a detailed review of health complications and major pathological mechanisms including mutation, inflammation, oxidative stress, and hemodynamic and plasma protein changes associated with chronic smoking. Further, we discuss about prophylactic interventions and associated benefits and provide a rationale and scope for clinical treatment.

Annual deaths of more than 480,000 in US due to cigarette smoking can be statistically categorized as follows: lung cancer ~ 29%, other cancers ~ 8%, ischemic heart disease ~ 28%, chronic obstructive pulmonary disease (COPD) ~ 21%, stroke ~ 4% and other diagnoses ~ 10% [1]. Further, for every person dying due to smoking, there are ~ 30 people with a severe smoking-related illness [2]. Out of the many deaths associated with CS, around 40,000 are due to second hand smoke (SHS) exposure [2]. Epidemiologically, nearly 16.7% adult men and 13.6% adult women smoke, and around 17% of them fall in the age group of 25–64 years. Ethnically, cigarette smoking is highest among non-Hispanic American Indians/Alaska Natives and people of multiple races, while it is the lowest among Asians.

Tobacco addiction and dependence make it important to ensure that both effective behavioral and pharmacological cessation treatments are made available to smokers who want to quit [3]. The Fagerstrom test for nicotine dependence helps understanding the severity of addiction. The higher the total Fagerström score, the more intense is the patient’s physical dependence on nicotine. Clinically, treatments targeting different aspects of nicotine addiction, such as reinforcement, withdrawal, and cue-associated learning ranging from complete cessation to substitution with less harmful products are enforced accordingly. Pharmacologic intervention such as nicotine replacement therapy include the use of both over the counter and prescription products like nicotine patch, gum, lozenges, inhalers and nasal sprays. Prescription non-nicotine medications such as bupropion (inhibits norepinephrine and dopamine reuptake) and varenicline tartrate (a partial α4β2 agonist) have also been found to be effective for quitting [3–6]. Counseling is often combined with medication and the combination has been found to be more effective for treating tobacco dependence than either medication or counseling alone. Smoking cessation reduces the risk of several associated health disorders. According to the Centers for Disease Control and Prevention (CDC), 1 year of smoking cessation significantly decreases the risk of heart attack and stroke. The risk for lung cancer drops by half, 10 years after quitting smoking [3–6]. However, many studies report that quitting smoking after 45 years of age does not have any beneficial health effect and does not decrease the risks for cardiovascular and other diseases associated with CS [7].

The Food and Nutrition Board of the National Academy of Sciences recommends a higher dietary allowance (RDA) of vitamin C for smokers (35 mg/day more compared to non-smokers) [8]. However, the health benefit and overall impact of this regimen on chronic smokers is still uncertain. As for the prophylactic treatment with other antioxidants, the results are quite controversial and highly dependent upon the experimental settings, purity of the agent/s and regimen of administration [9–13]. Laboratory studies have reported beneficial effects of a number of popular antioxidants and health supplements emphasizing their reactive oxygen species (ROS) scavenging and/or anti-inflammatory properties [14, 15] such as vitamins, resveratrol, melatonin, lipoic acid, etc. without taking into consideration their impact on the growth and proliferation of the cancerous cells [16]. In contrast, some clinical studies evaluating the health benefits of vitamins and antioxidants in smokers have shown mixed results including beneficial effects and instances where no changes were observed. These studies have been discussed in this paper. Unfortunately, no guidelines exist for clinical treatment for these patients (ex-smokers) or people exposed to SHS to alleviate the health impact of smoking. Clinical treatment only begins upon the manifestation of a challenging chronic disease such as cancer, diabetes and others.

This review paper discusses upon the health complications due to cigarettes smoking and the mechanisms involved in those pathophysiological complications. It further elaborates upon the current status of prophylactic interventions in smokers, thereby leading the discussion to the necessity and scope for therapeutic intervention in smokers including those exposed to SHS.

## Discussion

### Cigarette smoke composition

Tobacco smoke from a burning cigarette is a highly concentrated aerosolized collection of chemical particles ranging from aromatic amines, nitrosamines, aza-amines, ammonia, pyridine, acrolein to nicotine and many others. Addiction to tobacco smoking is primarily caused by nicotine. However, recent studies have also shown that non-nicotine components in tobacco such as anabasine, anatabine and norharmane have addictive properties (by acting as a monoamine oxidase inhibitor) on their own and can further reinforce that of nicotine. Tobacco smoke contains more than 7000 chemicals including 69 different carcinogens as well as a number of oxidative elements which can severely impact cells and tissue function and are prodromal to the onset of major health disorders [4, 5, 7].

Chemicals in cigarette smoke may be present in either particulate phase, the gas phase or a combination of both. The gas phase primarily contains sufficiently volatile chemical constituents such as hydrocarbons, nitrosamines, carbonyl compounds and gases such as nitrogen (N_2_), oxygen (O_2_), carbon dioxide (CO_2_), CO, hydrogen cyanide (HCN), hydrogen sulfide, acetaldehyde, methane, ammonia and others. The particulate phase contains water, phenols, humectants, carboxylic acids, terpenoids, paraffin waxes, catechols, polycyclic aromatic hydrocarbons (PAHs), tobacco-specific nitrosamines (TSNAs) and alkaloids such as nicotine, anatabine and others. In summary, combustion of a cigarette delivers toxic, carcinogenic and addictive compounds to the smokers [7].

### Health complications and major pathological mechanisms

Smokers in comparison to non-smokers are 2–4 times more likely to suffer from coronary heart disease and stroke and approximately 25 times more likely to develop lung cancer. Further, smoking has been associated with the onset of diabetes mellitus (DM), rheumatoid arthritis, pneumonia, asthma, blindness, hardening of the arteries, reduced fertility and impairment of the immune system leading to enhanced risk and progression of infections of all kinds. The risk of developing diabetes is 30–40% higher for smokers in comparison to non-smokers and the impact is dependent upon the number of cigarettes smoked. Smoking during pregnancy increases the risk of ectopic pregnancy, preterm delivery, stillbirth, low birth weight, orofacial clefts in infants and sudden infant death syndrome [4, 17].

Cigarette smoking is a prodromal risk factor for numerous cerebrovascular and neurological disorders including stroke, Alzheimer’s [18], depression [7, 18], cognitive impairment and vascular dementia [19]. The negative cerebrovascular and neurological impact of smoking is largely due to ROS generated upon tobacco smoking [20, 21], consequent inflammation [22] and blood–brain barrier (BBB) impairment [23]. As a matter of fact smoking during pregnancy impacts the cerebrovascular development in the fetus [4, 7].

**Fig. 1:**
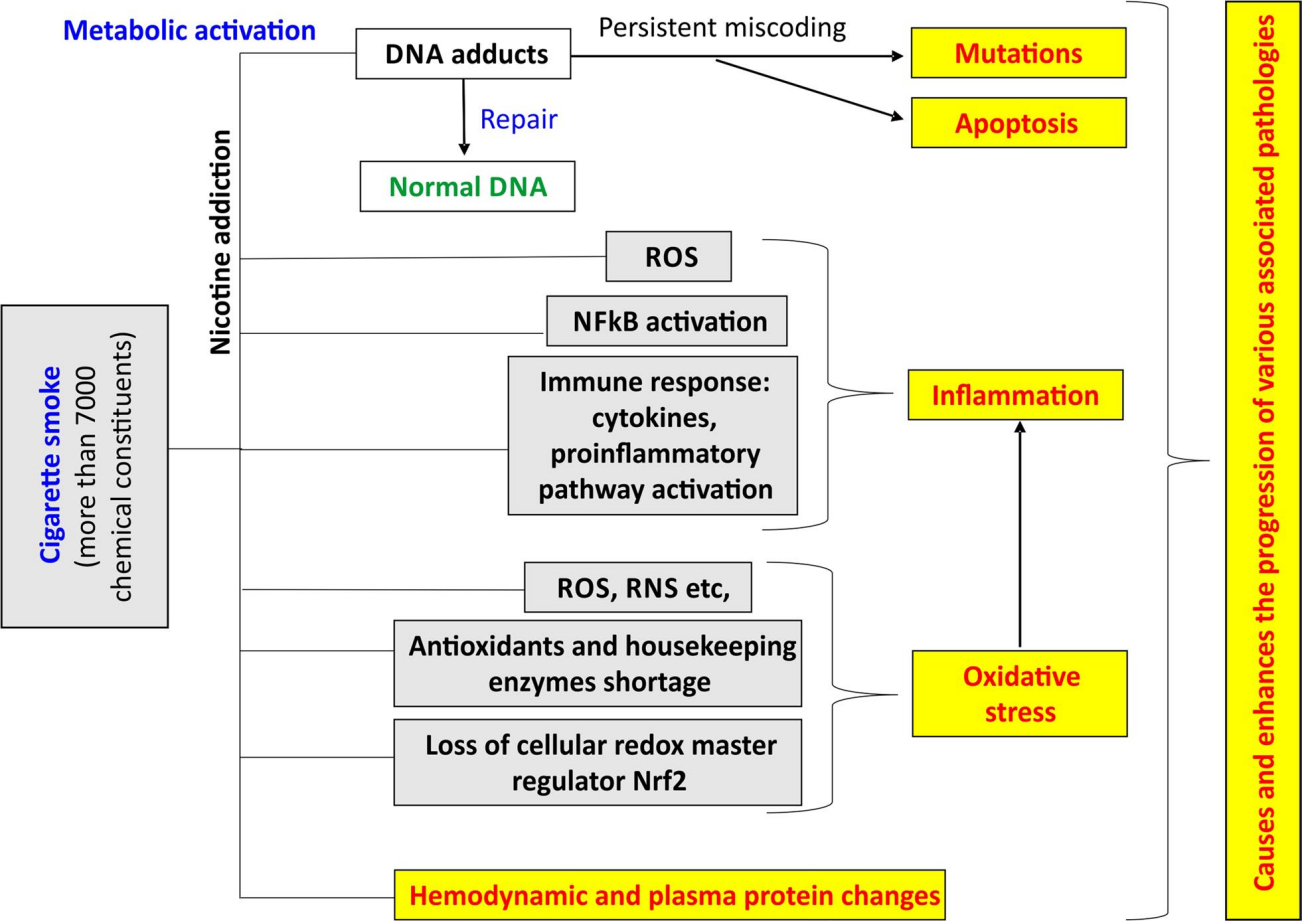
Proposed mechanisms for the toxicity observed due to cigarette-smoking. A combination of alterations/activation of various oxidation stress, inflammatory pathways and vascular changes accompany the beginning and progression of CS-induced vascular and cerebrovascular complications. Mutations in oncogenes play a major role in CS-induced cancers

The pathological impact of tobacco smoke involves four major mechanisms, namely mutation, inflammation, oxidative stress and hemodynamic changes, which we will elaborately discuss in the following topics (see also the schematic in Fig. 1).

- Smoking and mutation

Each puff of a cigarette contains several carcinogens belonging to multiple chemical categories such as PAHs, TSNAs, aromatic amines, metals, oxidants and free radicals that cause genotoxicity leading to eventual development of invasive cancers from healthy normal tissues [24]. These carcinogens undergo metabolic detoxification catalyzed by a variety of enzymes such as glutathione-S-transferases (GSTs), uridine-5′-disphosphate-glucuronosyltransferases (UGTs), epoxide hydrolases, and sulfatases. These carcinogens may also undergo metabolic activation by the action of P450 enzymes to forms that covalently bind to DNA and form DNA adducts. However, some carcinogens can form DNA adducts without any activation. These DNA adducts are regularly removed by the cellular repair mechanisms. Nevertheless, persistent formation of DNA adducts can cause miscoding which can eventually result in accumulation of permanent somatic mutations in critical genes (oncogenes and tumor-suppressor genes) leading to loss of normal growth mechanisms. The individual’s balance between the metabolic detoxification and activation of carcinogens vary and contributes to the differential pathological response in smokers with some developing cancer in just a couple of months while others taking years of chronic exposure to develop any pathology [7, 24, 25].

TP53 gene mutations have been reported in around 40% of human lung cancers and are observed to be more common in smokers in comparison to nonsmokers. On similar lines, KRAS gene mutation has been reported in approximately 30–40% of adenocarcinomas but rarely in squamous cell carcinoma, small cell lung cancer or tumors from non-smokers. TP53 gene acts as a tumor suppressor gene by sensing DNA damage and by playing a critical role in cell-cycle checkpoints, DNA repair, apoptosis and senescence. Studies have reported mutations in TP53 and other critical tumor suppressor genes such as RB, CHFR, MYO18B, PTEN and LKB1, resulting in a loss of their function, thereby leading to immortalization of bronchial epithelial cells. This is paralleled by activation of oncogenes such as KRAS leads to the disruption of several cellular pathways such as transcription, translation, cytoskeletal organization and cell–cell interactions, thus promoting neoplastic transformation [7, 24–26].

(b) Smoking and inflammation

Studies demonstrate that tobacco smoke activates proinflammatory/inflammatory pathways as evidenced by increased counts of circulating leukocytes and its adhesion to blood vessel walls, C-reactive protein, and acute-phase reactants such as fibrinogen [27–29]. These are due to both nicotine and other non-nicotine contents of the cigarettes. Studies report that nicotine acts as a chemotactic agent for migration of neutrophils besides increasing leukocyte adhesion to micro endothelium. Nicotine also acts by inducing the expression of a variety of proinflammatory cytokines such as Interleukins 6, 12 in variety of cells. Nicotine has also been reported to stimulate dendritic cells for enhanced proliferation of T cells and cytokines [7]. Further, these changes are augmented by the generation of ROS and other chemical forms resulting from the combustion of both nicotine and non-nicotine contents present in a cigarette. Immunologic mechanisms includes a greater Th2/Th1 ratio to increase the production of IgE, leading to greater allergic sensitization [30]. Lately, increasing evidence report T-helper 2 (Th2) cells activation upon smoke exposure and that it positively impacts the regulation of cytokines such as IL-4, IL-5, and IL-13 [31, 32]. These have been suggested to be responsible for increases in the frequency and severity of asthma-related exacerbations due to smoke exposure [33, 34].

An important mechanism by which smoking produces an inflammatory response is the activation of the NF-κB pathway which results in enhanced transcription of many genes involved in immune regulation. Activation of NF-κB by smoke also induces the protein expression of adhesion molecules besides promoting the migration of macrophages [35]. However, prolonged exposure to CS has been shown to have no effects on inflammatory mediators such as VCAM-1, ICAM-1 and cytokines in vivo [36, 37]. A decrease in endurance capacity and systemic inflammation has also been reported upon prolonged smoke exposure in mice [38]. Overall, the initial stimulation of inflammatory mechanisms followed by a loss of activation in immune responses correlates well with reports that smokers suffer from a compromised immunity and are highly susceptible to infections.

(c) Smoking and oxidative stress

Oxidants and electrophiles arising from internal metabolism and xenobiotic sources play an important role in maintaining physiological functions, cell signaling and cellular defense mechanisms. However, excessive generation of ROS by both internal (such as DM) and external factors (such as tobacco smoke—TS) initiates events such as anti-oxidant depletion, lipid peroxidation, and cellular toxicity thereby creating a state of redox imbalance [39]. A growing body of evidence indicates that oxidants and electrophiles are among the principal mediators involved in the initiation and progression of several vascular and cerebrovascular diseases, such as chronic inflammatory disease, stroke, neurodegenerative diseases such Alzheimer’s, Parkinson’s and amyotrophic lateral sclerosis (ALS), and aging [39–44]. Cells counteract ROS and oxidative stress by action of: (a) housekeeping enzymes such as catalase, superoxide dismutase, glutathione peroxidase; (b) direct antioxidants such as glutathione, ascorbic acid, tocopherols and; (c) indirect antioxidants consisting of a wide range of chemicals/natural agents that are capable of inducing cytoprotective responses [45]. Under normal conditions, ROS is scavenged or converted into less reactive molecules by the intracellular action of superoxide dismutase (SOD), catalase, glutathione (GSH) peroxidase [46] or (extracellular) antioxidant vitamins such as ascorbic acid (vitamin C), and α-tocopherol (vitamin E) [47–50]. However, both active and passive smoking can generate ROS beyond the levels which human body can effectively eliminate. Supporting this fact, several studies have shown that chronic smokers suffer from antioxidant shortage due to its increased mobilization to combat systemic oxidative stress evoked by ROS-enriched CS. Further, the oxidation and inflammation induced by CS in animals and cells are reduced on antioxidant supplementation [20, 51, 52]. A recent study in our lab demonstrated that CSE contains high concentrations of NO and hydrogen peroxide which corresponded with significant increase in cellular oxidative stress (measured using CellROX^®^ Green Reagent). Significant upregulation of culture medium level of proinflammatory cytokines (IL-6, MMP-2) was also observed [53]. These events can lead to potential oxidative damage to vascular system and the endothelium over a period of sustained exposure to CS (e.g., chronic smokers) and facilitate the pathogenesis and progression of vascular disorders [7].

**Fig. 2:**
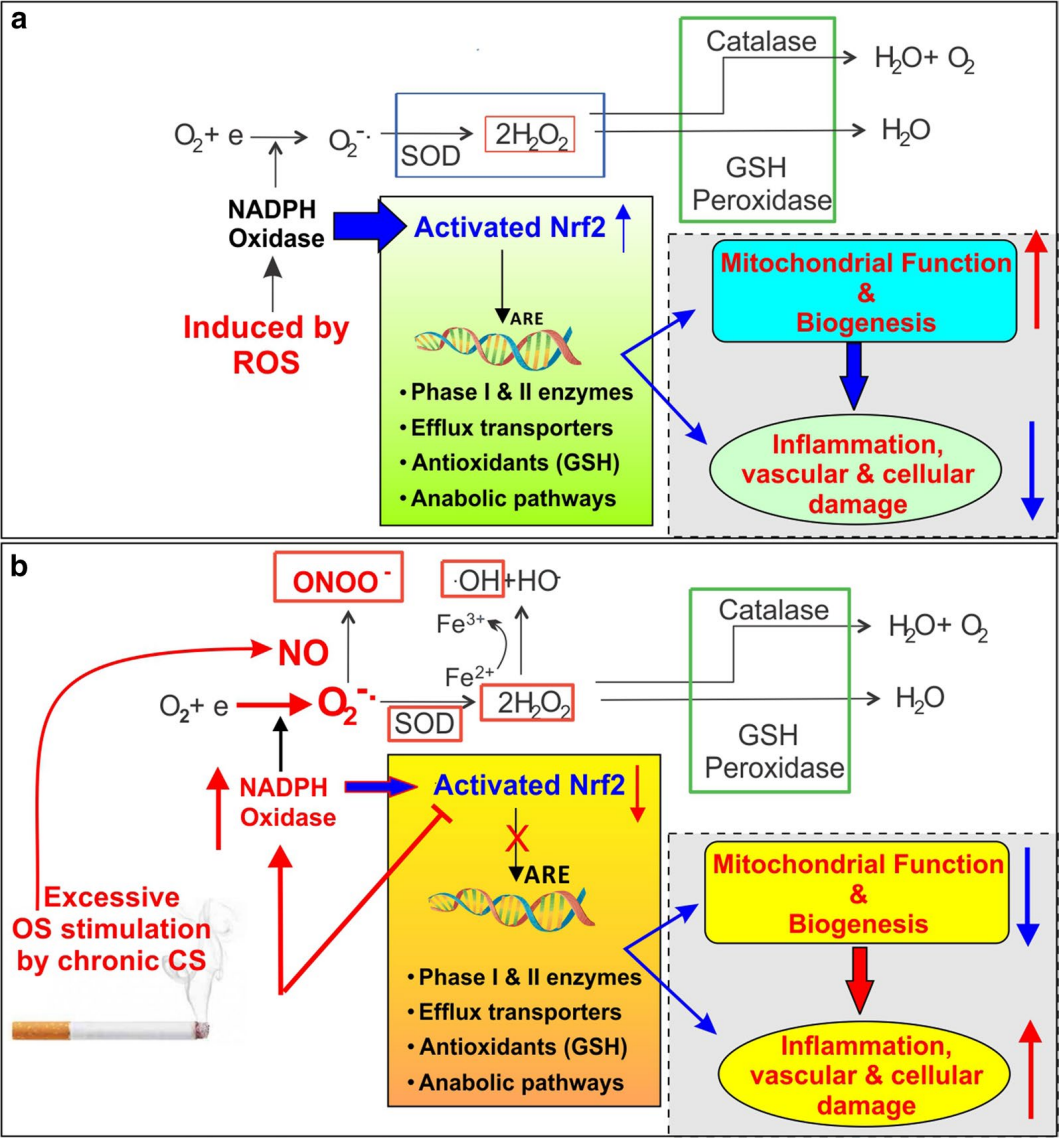
Activation of the cellular antioxidative response system under normal and stress condition. **a** Under normal conditions, the response to injury is adaptive, designed to restore homoeostasis and to protect the cell from further injury. **b** In response to excessive oxidative stress promoted by chronic CS exposure, NADPH oxidase is activated, producing an excess of O2– which in the presence of nitric oxide (.NO; also abundant in CS and release in response to IR) results in formation of peroxinitrite (ONOO–). Furthermore, the excess of H_2_O_2_ (not neutralized by catalase or GPx) leads to the formation of hydroxyl radicals (OH; Fenton’s reaction). The Nrf2-ARE system becomes dysfunctional leading to imbalances in mitochondrial redox homeostasis and biogenesis, inflammation, vascular and cellular damage which are all prodromal to a large number of CNS and systemic/peripheral pathologies

The DNA regulatory sequence accountable for stimulation by indirect antioxidant pathway inducers was identified as an antioxidant response element (ARE), while Nrf2 was subsequently recognized as the xenobiotic-activated receptor (XAR) major regulator responsible for the activation of ARE-dependent drug metabolizing enzymes [39]. In normal cells, Nrf2 is kept at low levels in cytoplasm by constitutive synthesis and degradation, which upon activation translocates into the nucleus and binds to the ARE loci. This results into the transcriptional activation of several antioxidant genes such as phase I/II detoxification (including cytochrome P450s), efflux transporters (classified as Phase III detoxificants) and antioxidant based systems involving glutathione, thioredoxin and others [54, 55] (see also Fig. 2). Nrf2-ARE pathway has been reported to be activated upon acute CS exposure, while its deficiency in mice leads to the development of extensive emphysemas following chronic CS exposure for 6 months [39]. A dysfunctional Nrf2 system (low levels, epigenetic changes and mutations) has been linked to elevated risk to develop both vascular diseases such as diabetes, cancer, COPD (regular and CS-induced) and chronic neurovascular diseases such as Parkinson’s disease (PD), Alzheimer’s disease (AD) and ALS [39, 42]. Our recent study in C57/BL6 mice exposed to CS for 1 month revealed significant downregulation in Nrf2 and its downstream protein NQO-1 levels in total brain homogenates [36], indicating a similarity in Nrf2 deficiency observed in lungs upon chronic CS exposure.

(d) Smoking, hemodynamic and plasma protein changes

Nicotine activates the sympathetic nervous system thereby causing an increase in blood pressure and heart rate. Cryer and colleagues observed increases in plasma epinephrine (around 150%, from 44 to 113 pg/mL) and norepinephrine levels (from 227 to 315 pg/mL) paralleled with positive chronotropic and inotropic changes in smokers soon after smoking. They further reported that these increases in heart rate and blood pressure were eliminated upon pretreatment with α and β-receptor blockers, validating the relation between smoking and sympathetic nervous system response [56]. Smokers in comparison to non-smokers have higher levels of triglycerides, very low density lipoprotein (VLDL), APO B, oxidative modification of LDL cholesterol (LDLc) and lower levels of high-density lipoprotein cholesterol (HDLc) and APO A-I [57–60]. These alterations in lipid metabolism are detrimental and support the progression of atherogenic dyslipidemia associated with cigarette smoking [7]. In addition to the effects of CS on the cellular elements of blood, smoking alters the protein levels of procoagulant and anticoagulation factors such as increased levels of fibrinogen; increased nitration of tyrosine residues on fibrinogen [29, 61], decreased thrombolysis [62] and decreased levels of thrombomodulin [36], thereby facilitating a prothrombotic state capable of inducing stroke or other hemorrhagic events.

### Prophylactic interventions and benefits

Antioxidants such as vitamins (vitamin C and E) [63, 64], β carotene [65, 66], coenzyme Q10 (CoQ10), melatonin, glutathione, lipoic acid, resveratrol [67] have shown to scavenge exogenous ROS in various experimental settings (cells and exposure conditions), implicating decrease in oxidative stress which is prodromal to risk and progression of several chronic diseases caused independently or due to CS. Besides acting as direct scavengers of ROS, both vitamin C and E have also been reported to reduce lipid peroxidation [68, 69], lymphocyte production, cytokine release, cellular adhesion molecule expression in monocytes [64, 70] and histamine release [71] due to CS exposure thereby acting as an anti-inflammatory agent. Five consecutive days of either vitamin C or E (100 mg/kg/day) pretreatment completely prevented DNA single strand breaks in the lung, stomach and liver of male ICR mice exposed to CS in comparison to controls [72]. Vitamin E treatment also increased the activity of antioxidant enzymes such as superoxide dismutase, catalase and glutathione peroxidase in CS exposed animals compared to untreated positive controls [73]. In another in vitro study, serum from eight smokers and non-smokers (age, gender matched with no other coronary risk factors) was added to confluent monolayers of human umbilical vein endothelial cells with and without l-arginine or vitamin C treatment for 24 h. Addition of l-Arginine reversed the increase in monocyte-endothelial cell adhesion due to CS. However, this inhibition in adhesion was not observed in cells upon vitamin C treatment [74].

Different degrees of protective effects were observed in an in vitro study where a comparative analysis was made between the popular antioxidants—CoQ10, melatonin, glutathione, lipoic acid and resveratrol in terms of decrease in the levels of cytokine release such as IL-6 and IL-8, proinflammatory adhesion molecule expression such as VCAM-1 and PECAM-1, monocyte adhesion and release of angiogenic factor VEGF. Overall, all the antioxidants decreased the increase in VEGF levels due to CS exposure and demonstrated anti-inflammatory effects [67]. Several beneficial effects of antioxidant supplementation against CS have mostly been reported both in vitro and in vivo. However, the translational significance of administering these antioxidants for therapeutic effects is the most important aspect where mostly controversial results arise.

An association between smoking and low levels of ascorbic acid (vitamin C) in serum has been reported which might be due to increased metabolic demand, elimination and decreased absorption of ascorbic acid. Oral supplementation with ascorbic acid has shown to raise its level in smokers. Despite its increased bioavailability, oral supplementation with ascorbic acid in several studies did not improve lipid peroxidation status in smokers [75]. Surprisingly, in a 2-month randomized, single-blind, placebo-controlled clinical trial, LDL oxidation actually worsened in smokers subsequent to supplementation [76]. However, another ascorbic acid supplementation study in smokers reported a significant decrease in urinary levels of 8-epi-prostaglandin (PG) F2 α (8-epi-PGF2α), a stable product of lipid peroxidation, suggesting a positive modulation of prostaglandin metabolites formed in the arachidonic acid pathway which can functionally compensate for the increased levels of free radical stress due to smoking [77]. Yet another study revealed no changes in urinary excretion rate of 8-hydroxydeoxyguanosine (8-OHdG), a repair product of DNA used as a biomarker to evaluate free radical damage, in smokers vs. non-smokers upon ascorbic acid, Vitamin E or CoQ10 supplementation [78].

A beta carotene and retinol efficacy trial (CARET) consisting of approximately 60% current smokers and 39% ex-smokers (total of 18,314 male and female subjects) exposed to asbestos, was conducted for 4 years to evaluate the therapeutic efficacy of either retinol (25,000 IU/day) plus β-carotene (30 mg/day) or placebo [79]. On similar lines, another Physicians Health Study (PHS), a long-term randomized, double-blind, placebo-controlled intervention trial designed to monitor the end points of cancer and cardiovascular incidence and mortality in 22,071 male physicians consisting of smokers and non-smokers upon beta-carotene or placebo administration was conducted [80]. Twelve years of supplementation with beta carotene produced neither benefit nor harm in the PHS study. Interestingly, the combination of beta carotene and vitamin A in the CARET study had no beneficial effects and may have had an adverse effect on the risk and death due to lung cancer, cardiovascular disease, and other causes in smokers and workers exposed to asbestos with no explanation for the possible adverse associations that were observed. Furthermore, these studies clearly negated the efficacy or safety of supplemental beta carotene or vitamin A in efforts to reduce the burdens of cancer or heart disease in certain populations especially smokers and recent ex-smokers [79, 80].

Unlike β-carotene and ascorbic acid which are reported to be low in the serum of smokers, literature suggests that smokers have equivalent [81, 82] or higher [83, 84] concentrations of α-tocopherol in comparison to non-smokers. Despite this similarity in α-tocopherol levels, smokers report higher tendency for lipid peroxidation [81, 84], suggesting a need for additional levels of vitamins in smokers to combat the increased levels of oxidative stress and its subsequent effects on lipid peroxidation. In the α-tocopherol, β-carotene cohort study (ATBC study) conducted in 29,133 male smokers, a 19% reduction in lung cancer incidence was observed in the highest versus lowest quintile of serum α-tocopherol [85]. The relationship between reduction in cancer incidence and α-tocopherol supplementation appeared stronger among younger persons and among those with less cumulative smoke exposure. However, a subsequent intervention study tracking the clinical end points of the ATBC study did not indicate an overall role of α-tocopherol in positively modifying clinical endpoints of cancer or heart disease in smokers. Later reports suggested alteration in metabolism and levels of several other antioxidant compounds after supplementation with pharmacological doses of α-tocopherol in smokers [84, 86–88]. With respect to cardiovascular disease, the α-tocopherol intervention resulted in fewer deaths from ischemic heart disease and ischemic stroke but an increased incidence and mortality from hemorrhagic strokes. Overall, the number of total strokes was not statistically different between the control and smoker group receiving α-tocopherol supplementation. In another study by Porkkala-Sarataho et al. in a population of male smokers with daily doses of 200 mg of RRR-α-tocopheryl acetate, no improvement in in vivo plasma levels of malondialdehyde (MDA) was noted. However, an increase in vitro lag time was noted, suggesting increased ability of LDL to resist oxidative stress. The combined supplementation of vitamin E and C (in a 36 month follow up) increased the oxidation resistance of total serum lipids more efficiently than either supplementation alone [89].

Overall, clinical trials with vitamins have shown mixed results in decreasing the pathological incidences associated with smoking. Nevertheless, the increase in capability of individuals to combat oxidative stress has been seen. These studies have mostly evaluated certain biomarkers such as metabolites of nicotine or in vitro assessment of lipid peroxidation and clinical parameters which might not be the ideal way to assess the beneficial effects. What about the improvement in the quality of health in ex-smokers upon the supplementation? Lifespan increase and decrease in incidence of pathological conditions does not necessarily indicate improved health of an individual. Other parameters such as decreased susceptibility to infections or increased expression of major transcription factors such as NF-κB and Nrf2 in RBCs or platelets collected during such clinical trials can better reflect the anti-oxidative capacity of the cells. It might be possible that vitamins and other anti-oxidants are capable of combating exogenous ROS, but are incapable in overcoming the cellular damage caused due to chronic smoking.

### Rationale and scope for clinical treatment

Strangely, immunocompromised diseases such as asthma and other chronic ailments such as diabetes and major cancer took a steep rise in its occurrence in the twentieth century after the prevalence of smoking, alcohol and sedentary lifestyle came in. Although tobacco consumption existed from ancient times, its consumption in the form of cigarettes became popular after the invention of the automated cigarette making machine in 1881 by James Bonsack [90]. As discussed earlier, tobacco smoke can damage each and every part of the human body primarily due to mutation, inflammation, oxidative stress and other vascular changes [7]. Sadly, there is no available clinical treatment for these smokers that can prevent the occurrence of the metabolic changes further leading to various pathologies. Both active and passive smoking causes glucose intolerance [91] besides major pathological changes such as insulin resistance and high levels of glycated hemoglobin (HbA1c) [92] as reported in diabetic patients. On similar lines, proof-of-concept experiments have demonstrated that Nrf2 (major redox transcription factor) deficient mice developed early and extensive emphysema upon chronic CS exposure for 6 months [93] and worsened the diabetic phenotype in mice [94]. Further, the synthetic triterpenoid 1[2-cyano-3,12-dioxooleana-1,9(11)-dien-28-oyl]imidazole (CDDO-Im), a known Nrf2 inducer, significantly reduced lung oxidative stress, alveolar destruction and emphysema caused by chronic CS exposure [95].

A cohort study of 551 patients with systolic heart failure (HF) was evaluated for all-cause mortality or urgent transplantation to understand the impact of statin therapy in these patients. About 73% of the total cohort, 80% of the statin treatment group and 66% of non-treatment group consisted of patients with smoking history. Statin therapy was found to be associated with significantly improved survival free from urgent transplantation (84% in statin-treated and 70% in non-treated patients) in statin treated group comprising of patients with higher rates of hypertension, diabetes, and smoking in comparison to controls (left ventricular ejection fraction and cholesterol levels were similar between treated and non-treated patient groups) [96]. Another cohort study evaluating the efficacy of statin treatment on risk of coronary heart disease in patients with familial hypercholesterolaemia was conducted in 2146 patients with familial hypercholesterolaemia without prevalent coronary heart disease. In this study too, about 77% of treatment group and 72% of the non-treatment group had a smoking history. Results indicated that lower statin doses than those currently advised reduced the risk of coronary heart disease in patients with familial hypercholesterolaemia [97]. These beneficial effects might be due to the anti-atherothrombotic and anti-inflammatory actions of statins. Further stains have also known to modulate pathologic ventricular remodeling and angiotensin II signaling besides normalizing sympatho-excitation associated with heart failure [96].

On similar lines, metformin (MF) use in diabetic patients has been associated with lesser risks of cancer occurrence [98, 99]. Molecular studies both in vitro and in vivo have also shown that metformin treatment ameliorates the adverse effects of CS toxicity at cerebrovascular level and BBB endothelial cells. MF not only prevented the breakdown of tight junction proteins such as ZO-1 and Occludin but also was observed to negate the decrease in Nrf-2 and Glut-1 levels, thereby restoring the metabolic and redox balance of the cells [36]. Metformin was initially reported to act through activation of 5′ AMP-activated protein kinase (AMPK) related pathways. However, numerous studies have also stated many of its beneficial effects to be independent of AMPK activation, highlighting its therapeutic potential in context of several other health challenges and diseases such as cardiovascular diseases, cancer and ageing [100]. Metformin has been recently reported to promote neurogenesis and enhance spatial memory formation indicating its therapeutic value for the injured or degenerating neurovasculature [101]. Furthermore, MF has also been shown to attenuate BBB disruption and decrease/inhibit ischemic injury upon stroke via AMPK dependent and independent (Nrf2 antioxidant pathway) mechanisms [102, 103]. A recent published work by our group revealead that MF drastically reduces the brain and cerebrovascular toxicity of TS while also protecting BBB integrity through the activation of Nrf2 [36]. Additional in vivo evidence shows that MF effectively reduces the risk for stroke and attenuates post-ischemic brain injury promoted by TS and e-Cig vaping. As such, MF could be used in the prophylactic care treatment to renormalize the risk levels of stroke immediately following smoking cessation, thus warranting further studies in that direction [104].

Based on these premises, further research studies focused on clinical treatment for smokers, ex-smokers and SHS is warranted. Health supplements have shown some beneficial effects, however, their use is rarely advised by physicians other than for general wellbeing and support of hormonal balance.

In addition, omic technologies may enable the identification of non-invasive markers for early identification of smokers at higher risk for tobacco-induced lung damage, thus enabling the development of preventive therapeutic strategies [1, 105–108].

## Conclusion

From a translational and clinical point of view, studies clearly support the idea that strategies aimed at restoring metabolic and redox activity hold tangible therapeutic potential to reduce the burden of CS not only in chronic smokers but also in early stage former smokers and SHS who are still at high risk of developing vascular, cerebrovascular disorders and more severe secondary brain injuries than life-long non-smokers. Clinical treatment for smokers holds viable promises. However, there are several challenges towards it, namely: (1) further in-depth understanding of mechanisms involved in CS-induced vascular and cerebrovascular impairments; (2) pre-clinical and clinical evaluation of therapeutic drugs in preventing secondary level injury such as cerebrovascular stroke upon CS exposure; (3) identification of vascular markers in smokers, ex-smokers and second hand smokers that indicate a significant level of oxidative stress to begin clinical regimen and; (4) identification of conditions such as epigenetic mutations in smokers (Nrf2 mutation has been linked to progressive growth of cancerous cells upon further stimulation) that indicate their exclusion from clinical treatment. Genetic variability in the basal expression of these anti-oxidant genes may provide a plausible explanation in the wide difference between people’s responses to smoking. As discussed earlier, carcinogens present in CS cause mutations. Mutations in these transcription factors specifically Nrf2 has been implicated in robust proliferation of cancerous cells [109]. For such situations, newly developing technologies such as gene editing holds substantial promises.

## Abbreviations

AD: Alzheimer’s disease
ALS: amyotrophic lateral sclerosis
AMPK: 5′ AMP-activated protein kinase
ARE: anti-oxidant response element
ATBC: α-tocopherol, β-carotene cohort study
BBB: blood–brain barrier
CARET: beta carotene and retinol efficacy trial
CDC: Centers for Disease Control and Prevention (CDC)
CDDO-Im: 1[2-cyano-3,12-dioxooleana-1,9(11)-dien-28-oyl]imidazole
COPD: chronic obstructive pulmonary disease
CoQ10: coenzyme Q10
CS: cigarette smoke
DM: diabetes mellitus
GSH: glutathione
GSTs: glutathione-S-transferases
HF: heart failure
HDLc: high-density lipoprotein cholesterol
LDLc: LDL cholesterol
MDA: malondialdehyde
MF: metformin
Nrf2: nuclear factor erythroid 2-related factor
PD: Parkinson’s disease
PG: prostaglandin
PAHs: polycyclic aromatic hydrocarbons
PSH: Physicians Health Study
RDA: recommended dietary allowance
ROS: reactive oxygen species
SHS: secondhand smoke
SOD: superoxide dismutase
Th2: T-helper 2
TS: tobacco smoke
TSNAs: tobacco-specific nitrosamines
TTC: triphenyl tetrazolium chloride
XAR: xenobiotic-activated receptor
UGTs: uridine-5′-disphosphate-glucuronosyltransferases
VLDL: very low density lipoprotein
